# A venom peptide-induced Na_V_ channel modulation mechanism involving the interplay between fixed channel charges and ionic gradients

**DOI:** 10.1016/j.jbc.2024.107757

**Published:** 2024-09-10

**Authors:** Ashvriya Thapa, Jia Hao Beh, Samuel D. Robinson, Jennifer R. Deuis, Hue Tran, Irina Vetter, Angelo Keramidas

**Affiliations:** Institute for Molecular Bioscience, The University of Queensland, St Lucia, QLD, Australia

**Keywords:** sodium channel, peptides, pain, neurotoxin, channel activation, biophysics

## Abstract

Venoms are used by arthropods either to immobilize prey or as defense against predators. Our study focuses on the venom peptide, Ta3a, from the African ant species, *Tetramorium africanum* and its effects on voltage-gated sodium (Na_V_) channels, which are ion channels responsible for the generation of electrical signals in electrically excitable cells, such as neurons. Using the Na_V_1.7 isoform as our model Na_V_ channel we show that Ta3a prolongs single channel active periods with increased open probability and induces non-inactivating whole-cell currents. Ta3a-affected Na_V_1.7 channels exhibit a leftward (hyperpolarizing) shift in activation threshold, constitutive activity even in the absence of an activating voltage stimulus, and at cell membrane voltages where channels are normally silent. Current-voltage experiments show that Ta3a shifts the voltage at which Na_V_ current changes direction (reversal potential) by altering the local ionic concentration of permeant ions (Na^+^) rather than changing the channel’s preference for ionic species. We propose a model where Ta3a maintains the positively charged voltage-sensing (S4) domains of the channel in the activated configuration where their electric field is exposed to the extracellular membrane surface to create an ionic bilayer comprising S4 domains and mobile anions (Cl^−^). This bilayer has a depolarizing effect on the cell membrane, thus reducing the amount of externally applied voltage required for channel activation.

Voltage-gated sodium (Na_V_) channels generate the rapid and brief upstroke of action potentials in electrically excitable cells. Within only a few milliseconds, Na_V_ channels cycle between resting, open (conducting), and inactivated states (1, 2, 3). These rapid functional transitions correspond to relative movements of structural elements of the channel that underlie the process of channel gating (4, 5).

Each Na_V_ channel consists of a single protein comprising approximately 1900 amino acids (6, 7) that is organized into four homologous domains (DI-DIV). These arrange around a central permeation pathway (4). Each domain consists of six transmembrane helices (S1-S6). S5, S6, and the re-entrant P-loop that connects them form the pore domains, which together assemble to form the Na^+^-selective pore. The S1-S4 helices encircle the pore domains and form the voltage-sensing domains. Notably, the four S4 helices, which contain positively charged residues (arginines or lysines) at 3-residue intervals, detect changes to transmembrane voltage and respond by moving relative to the membrane. Membrane depolarization initiates the gating process, which involves the S4 domains sliding obliquely from the “down” resting position where the charges are shielded from the extracellular compartment by the membrane, to the “up” activated position where they move across the electric field of the membrane and splay radially around the open, conducting pore (4, 5, 8, 9). This process exposes these gating charges to the extracellular space (8, 10, 11, 12). Channel inactivation has been mostly attributed to the upward translocation of the S4 helix of DIV, which engages a motif located within the DIII-DIV intracellular linker (2, 3, 5, 13) and two closely spaced hydrophobic rings of residues contributed by the S6 domains that are located at the intracellular mouth of the pore (14).

Venom-derived peptides and small molecules that interact with Na_V_ channels have provided a wealth of insight into their structure and function (4, 15, 16). By circumventing voltage-elicited channel activation, direct channel activators (agonists) such as veratridine and batrachotoxin have illuminated mechanisms of channel activation and ion permeation (15, 17, 18). Peptide toxins, such as the disulfide-rich peptides derived from arachnid venoms, exhibit channel state-dependent affinities at Na_V_ channels and generally potentiate or inhibit Na_V_ channel currents by affecting channel activation or inactivation, respectively. For instance, β-scorpion toxins enhance activation by stabilizing the DII S4 helix in the activated state (19), whereas the tarantula toxins, ProTx-II and huwentoxin-IV, inhibit Na_V_ channel currents by stabilizing S4 helices in the resting state (20, 21).

In this study, we investigated the effects of a recently described peptide, Ta3a, from the venom of the ant *Tetramorium africanum* (22), at the Na_V_1.7 channel isoform using whole-cell and single ion channel electrophysiology. Unlike the disulfide-rich peptides derived from arachnid venoms, Ta3a is cysteine-free and predicted to be α-helical in structure. Ta3a induced a pronounced leftward shift in the potential where current changes direction across the cell membrane (reversal potential, V_rev_) in addition to a leftward (hyperpolarizing) shift in the voltage threshold for channel activation (V_1/2_). Remarkably, Na_V_-mediated currents in the presence of Ta3a persisted even at extreme negative membrane potentials.

Our data are consistent with Ta3a stabilizing the positively charged S4 helices in the activated configuration to produce hyperactive Na_V_1.7 channels that display markedly prolonged single channel active periods and impaired whole-cell channel inactivation (22). The extracellularly exposed S4 segments effectively introduce a layer of fixed extracellular surface charges that induce cell membrane depolarization by modulating extracellular local mobile ion concentrations and membrane surface potential. Our study delineates a unique constellation of peptide-induced Na_V_ channel modulatory mechanisms that involves the interplay between fixed channel charges and ionic gradients that reduce the amount of externally applied depolarization required for channel activation (1, 23).

## Results

### Ta3a shows activity at multiple Na_V_ channel subtypes

**Figure 1:**
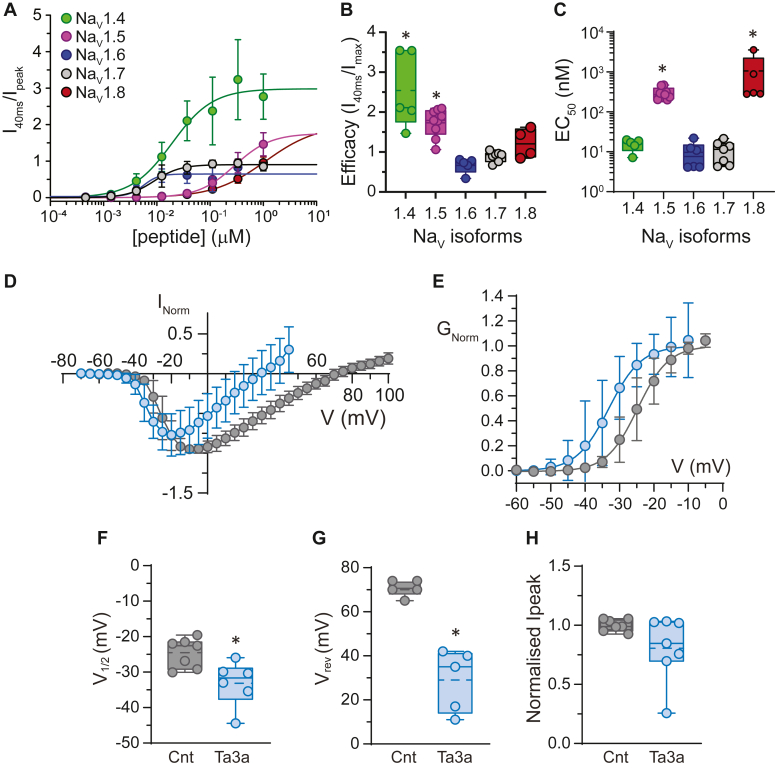
**Ta3a is active at Na**_**V**_**channel isoforms, shifts the current-voltage plot to the left and impairs whole-cell inactivation of Na**_**V**_**1.7 channels.***A*, whole-cell cumulative concentration-response plots of the indicated Na_V_ channel isoforms expressed in HEK293 cells over a range of Ta3a concentrations. Currents were elicited by depolarizing the cell membrane from −90 mV to −10 mV (for Na_V_1.4 to Na_V_1.7) or to +10 mV (for Na_V_1.8) for a duration of 50 ms. The plotted data represent the ratio of current amplitude at 40 ms after the onset of the activating voltage step and the peak current (I_40ms_/I_max_). *B*, Box and whisker plots of the efficacy with which Ta3a elicited non-inactivating currents for the indicated Na_V_ channel isoforms. *C*, Box and whisker plots of the potency (EC_50_) of Ta3a at the indicated channel isoforms. ∗*p* < 0.05 (one way ANOVA), n = 4 to 9 cells per Na_V_ channel subtype. *D*, current-voltage (I-V) plot recorded in standard extracellular solution showing the effects of Ta3a (30 nM, *blue*) on Na_V_1.7 channels expressed in HEK293 cells. The activating voltage protocol involved stepping the voltage from a holding potential of −90 mV followed by a series of 50 ms step pulses from −110 mV to +80 mV at 5 mV increments (repetition interval 6 s). Note the leftward shift in the I-V and the decrease in peak current in Ta3a-affected cells. *E*, conductance-voltage plot of the data in (*D*) showing a −8.5 mV leftward shift in the V_1/2_ in Ta3a-affected cells. *F*, Box and whisker plot showing the leftward shift in V_1/2_ in cells perfused with Ta3a. *G*, Box and whisker plot showing the change in reversal potential of −42 mV in cells perfused with Ta3a. *H*, Box and whisker plot showing peak currents in control and after Ta3a perfusion. ∗*p* < 0.05 (unpaired *t* test), n = 5 to 7 cells per plot. Data are expressed as mean ± SD. Na_V_, voltage-gated sodium.

We tested the potency (EC_50_) and the efficacy (maximum response) with which Ta3a induced persistent (non-inactivating) current by taking the ratio of current at 40 ms after an activating voltage step and the peak current (I_40ms_/I_peak_) at several isoforms of human Na_V_ channels. These experiments included representatives of tetrodotoxin (TTX) sensitive (Na_V_ 1.7, 1.6 and 1.4) and TTX resistant (Na_V_ 1.5 and 1.8) channels. Cumulative concentration-response experiments were done using a voltage step from −90 mV to −10 mV (50 ms duration). The concentration-response experiments demonstrate that Ta3a enhances persistent currents relative to peak of all the isoforms tested (Fig. 1*A*). Ta3a was more efficacious at Na_V_1.4 (2.5 ± 1.0, ANOVA, *p* = 0.004, n = 5 cells) and Na_V_1.5 (1.7 ± 0.4, *p* = 0.022, n = 9 cells) channels relative to our reference, Na_V_1.7 channel (0.89 ± 0.13, n = 7 cells). Similar efficacies were obtained for Na_V_1.6 (0.67 ± 0.19, *p* = 0.070, n = 5 cells) and Na_V_1.8 (1.22 ± 0.37, *p* = 0.116, n = 4 cells) relative to that of the Na_V_1.7 channel (Fig. 1, *A* and *B*). The EC_50_s of Ta3a were lower for TTX sensitive Na_V_1.7 channels (11.1 ± 6.5 nM), Na_V_1.4 channels (15.5 ± 5.1 nM, ANOVA, *p* = 0.417) and Na_V_1.6 channels (9.6 ± 7.1 nM, ANOVA, *p* = 0.892). Higher EC_50_s were obtained for the TTX insensitive Na_V_1.5 channels (297 ± 102 nM, ANOVA, *p* = 0.006) and Na_V_1.8 channels (1060 ± 1416 nM, ANOVA, *p* = 0.003) (Fig. 1, *A* and *C*) compared to the Na_V_1.7 channel. We chose the Na_V_1.7 channel as our model for the rest of the study because of its relatively high Ta3a sensitivity, and because it is a molecular mediator of pain transmission (22).

### Ta3a shifts the current-voltage (I-V) relationship of Na_V_1.7 channels toward negative voltages and reduces peak current

In response to a series of depolarizing voltage steps in the presence of 30 nM Ta3a, we observed three salient changes in the whole-cell current-voltage (I-V) relationship of Na_V_1.7 channels relative to control. First, a hyperpolarizing shift in the threshold for channel activation from a mean V_1/2_ (voltage required for half-maximal current response) of −24.5 ± 4.1 mV to −33.2 ± 6.4 mV (ΔV_1/2_ of −8.7 mV, unpaired *t* test, *p* = 0.0133, n = 6 cells, Fig. 1, *D*–*F*). Second, a leftward shift to the reversal potential, from 70.6 ± 3.7 mV to 29.0 ± 14.1 mV (ΔV_rev_ = −41.6 mV, unpaired *t* test, *p* = 0.0058, n = 5 cells, Fig. 1, *D* and *G*). Third, consistent reduction in peak current to 81.0 ± 0.3% of control (unpaired *t* test, *p* = 0.078, n = 7 cells Fig. 1, *D* and *H*). From these initial observations we hypothesized that Ta3a reduced V_1/2_ by affecting the membrane voltage or voltage sensing elements of the channel, and either alters the ionic selectivity of the channels or the transmembrane gradient of permeant ions to affect V_rev_. The reduction in peak current could be due to a reduced driving force for inward current but is not likely due to a reduced channel open probability or channel block because this would not be consistent with a leftward shift in V_1/2_. Alternatively, the reduction in peak current could reflect a channel population effect as a function of exposure time to Ta3a. To distinguish between these possibilities and test our hypotheses we carried out additional whole-cell experiments and single ion channel measurements.

### Ta3a induces persistently active (non-inactivating) channels

To investigate the underlying mechanisms affecting the I-V relationship when cells are perfused with Ta3a, we conducted separate whole-cell I-Vs in simplified extracellular and intracellular solutions where the only charge carriers were Na^+^ and Cl^−^ (24). The advantage of using simplified solutions is that hypothesized peptide-induced changes to ionic gradients that might alter the net driving force for current or any changes to ionic selectivity can be attributed to the flux of either Na^+^ or Cl^−^ ions, thus reducing the ionic variables in the standard model for selective ion permeation as described by the Goldman-Hodgkin-Katz equation (GHK, see Equations 2 and 3).

**Figure 2:**
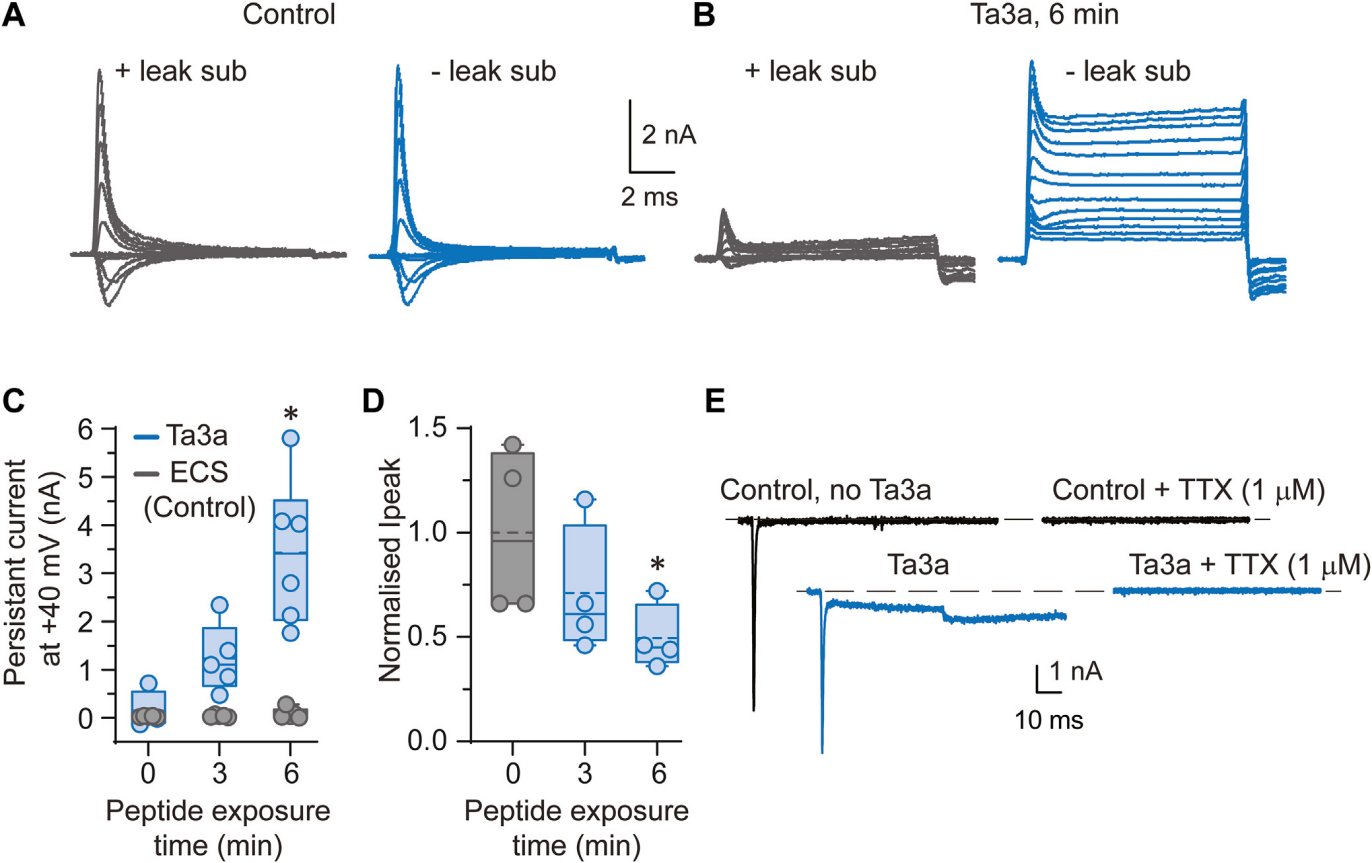
**Ta3a diminishes peak currents and enhances non-inactivating currents of NaV1.7 channels.***A*, representative control whole-cell currents in response to a series of depolarizing voltage steps showing recordings with and without leak subtraction. *B*, whole-cell currents in response to the same voltage steps as in (*A*) showing that Ta3a reduces the proportion of channels that can be activated by voltage steps and increases the proportion of channels that do not inactivate. Note the total current is the same without leak subtraction between (*A*) and (*B*). All currents in (*A*) and (*B*) were recorded in simplified NaCl containing intracellular and extracellular solutions and are from the same cell. Currents were elicited by a series of 50 ms voltage steps from a holding voltage of −110 mV to −70 mV and then at 10 mV (or 5 mV) increments to a final level of +100 mV. *C*, Box and whisker plot showing the time-dependent effects of Ta3a on persistent (non-inactivating) current compared to vehicle (no Ta3a) controls. *D*, Box and whisker plot showing the time-dependent decrease in peak currents in the presence of Ta3a. ∗*p* < 0.05 (one-way ANOVA), plotted data from 4 to 6 cells. *E*, representative whole-cell currents showing that the open Na_V_ channel blocker, TTX (1 μM, n = 4 cells) blocks both peak and the Ta3a induced non-inactivating current. Data are expressed as mean ± SD. Na_V_, voltage-gated sodium; TTX, tetrodotoxin.

These currents were monitored with and without leak subtraction to determine if the observed reduction in peak current could be accounted for by a steady-state persistent (“leak”) current that remained well after the channels normally inactivate. This experiment would also provide insight as to whether Na_V_ channels were blocked or inhibited in the sustained presence of Ta3a. Prior to applying Ta3a, the currents with and without leak subtraction in the same cell were indistinguishable (Fig. 2*A*). After the recorded cell was exposed to 30 nM Ta3a for 3 min, the reduction in currents became apparent only when leak subtraction was engaged. However, without leak subtraction the overall magnitude of the current was similar to control suggesting that the number of active channels remained constant throughout the experiment. This finding argues against channel block or inhibition by Ta3a. Notably, the persistent currents at all applied voltages did not return to baseline as was observed in the controls, suggesting that channel inactivation was impaired in the presence of Ta3a and could not be overcome by returning to hyperpolarized voltages. This pattern continued with longer Ta3a exposure time. At 6 min the peak currents diminished further, and the persistent currents increased, and here again, the total current amplitude remained constant throughout the recordings (Fig. 2*B*). Our simplified solutions produced significant outward currents, and at 6 min of Ta3a exposure the non-inactivating currents were outward for all applied voltages when leak subtraction was not engaged (Fig. 2*B*, *left*). This observation provides the first experimental indication that the ionic driving force for outward current had increased markedly in the presence of Ta3a. The exposure time dependent persistent current was plotted for currents obtained at +40 mV at 4 ms after current onset. Prior to Ta3a application, the persistent current was absent (147 ± 386 pA with no leak subtraction, n = 4 cells), but began to increase at 3 min of Ta3a exposure (1232 ± 707 pA, ANOVA, *p* = 0.2549, n = 5 cells) and further increased at 6 min of Ta3a exposure (3431 ± 1503 pA, ANOVA, *p* = 0.001, n = 6 cells, Fig. 2*C*). In a separate set of control cells expressing Na_V_1.7 channels that were perfused with extracellular solution alone, the persistent current was absent (Fig. 2*C*, n = 4 cells), demonstrating that the persistent current was induced by Ta3a. The peak inward current over the course of the experiment was also plotted and shows a time-dependent decrease in the presence of Ta3a to 71 ± 3% of control at 3 min (ANOVA, *p* = 0.231, n = 4 cells) and to 50 ± 2% of control at 6 min (*p* = 0.033, Fig. 2*D*). As additional confirmation that the non-inactivating component of the currents was indeed mediated by the Na_V_ channels, we applied the open channel blocker, TTX (1 μM), after exposing recorded cells to Ta3a. As illustrated in Figure 1*E* and consistent with our previous finding (22), in response to an activating voltage step before and after Ta3a exposure, both peak and non-inactivation components of the currents were completely ablated by TTX (n = 4 cells). These results strongly suggest that Ta3a over the course of the recordings bound to and persistently activated an increasing number of available channels, leaving a diminishing pool of channels that activated in response to externally applied depolarizing voltages. We attribute the observed decrease in peak current in our I-Vs, in part, to the exposure-dependent decrease in the number of channels that can be activated by standard voltage depolarization.

**Figure 3:**
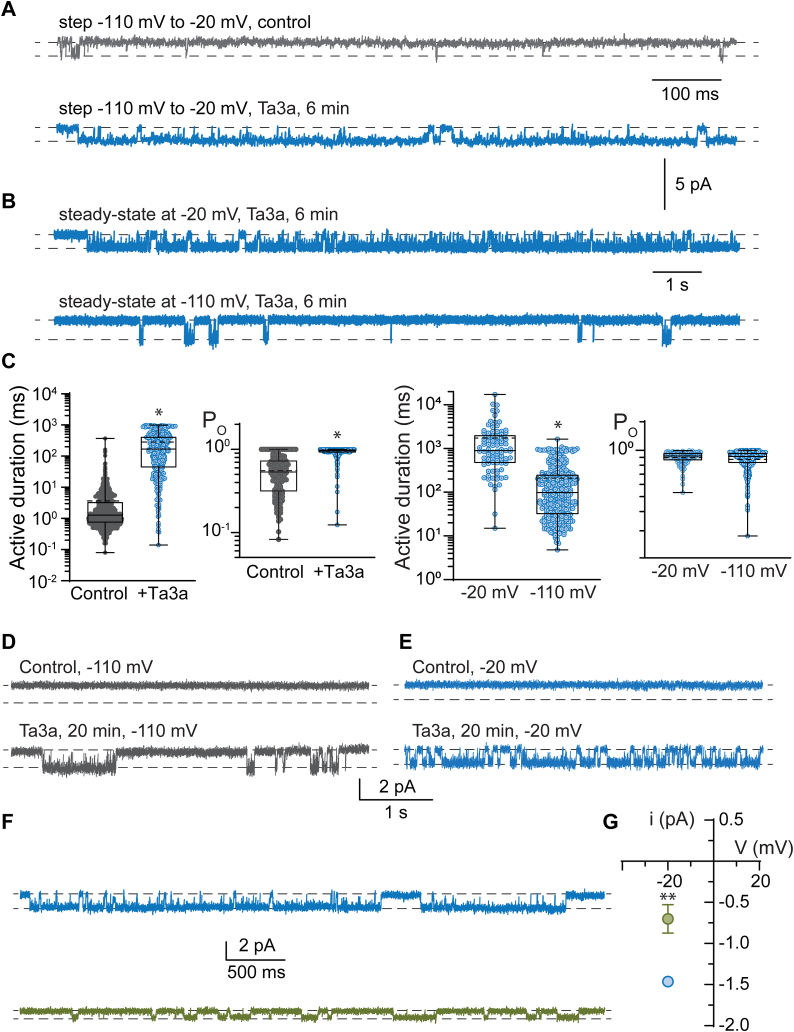
**Ta3a induces long single-channel activity and enhanced steady-state open probability (P**_**O**_**).***A*, representative single channel currents in response to a step in transmembrane voltage from −110 mV to −20 mV for 1 s duration in control conditions (*gray*) and after 6 min of continuous perfusion with Ta3a (30 nM) (*blue*). *B*, representative single channel currents after 6 min of Ta3a perfusion held at steady-state voltages of −20 mV and −110 mV after previous depolarizing steps. *C*, quantification of the single-channel active duration and P_O_ in control and Ta3a exposure in response to voltage steps (left pair) and steady state voltages (right pair). ∗*p* < 0.05 (unpaired *t* tests), n = 5 to 9 patches. *D* and *E*, single-channel recordings showing channel activity after exposure to Ta3a (30 nM) for 20 min in patches that were voltage clamped at the indicated voltages but never voltage stepped. *F*, single channel recordings after 3 to 5 min (*blue*, above) of continuous perfusion of Ta3a and about 20 min (*green*, below) after direct perfusion was stopped. *G*, the group data are plotted on the right (*p* < 0.005, paired *t* test, n = 3 patches).

To further confirm that the Ta3a-induced persistent, non-inactivating current was mediated by the Na_V_1.7 channels and to explore its voltage-dependence, we performed single-channel recordings in excised outside-out membrane patches, initially in response to voltage steps from −110 mV to −20 mV for 1 s duration. No channel activations were observed at the initial holding potential of −110 mV. However, upon depolarizing the membrane patch to −20 mV, clear single channel activations (periods of activity due to one ion channel) were observed. Multiple, 1 s recording periods at −20 mV elicited channel activity that occurred most frequently at the onset of the voltage change followed by longer quiescent periods corresponding to channel inactivation (Fig. 3*A*). Na_V_ channels occasionally reactivated for brief periods before the end of each 1 s sweep, and ∼5% of sweeps failed to produce any channel activity, likely reflecting channel inactivation from resting configurations (3).

After completion of the control recordings, 30 nM Ta3a was perfused continuously over each recorded patch. Channel activity was monitored by applying the same voltage step protocol as for the control recordings. Discrete channel activations became increasingly longer and saturated at a Ta3a exposure time of about 6 min (Fig. 3*A*).

While recording single channel currents in responses to voltage steps from −110 mV to −20 mV we noticed clear channel openings at −110 mV after exposure to Ta3a. This observation prompted us to explore whether Ta3a elicited Na_V_1.7 channel activity in the absence of a depolarizing voltage step. We recorded single-channel currents at steady-state voltages of −110 mV and −20 mV, after the patches had already been exposed to Ta3a and voltage-stepped from −110 mV to −20 mV (Fig. 3*B*).

We selected the Ta3a exposure time of 6 min to analyze Ta3a-induced activity further. Single-channel activations were analyzed for duration and intra-activation open probability (P_O_). In the absence of Ta3a, single Na_V_1.7 channels activated for a mean of 3.7 ± 0.4 ms (median, 1.28 ms, 1321 individual activations from five patches) (Fig. 3*C*). After 6 min of Ta3a exposure, the duration of single channels activity increased to a mean of 284 ± 294 ms (median, 168 ms, 315 individual activations, paired *t* test, *p* < 0.001) in response to voltage steps. The intraactivation P_O_ also increased from a control value of 0.56 ± 0.26 (median, 0.54) to 0.93 ± 0.11 (median, 0.97, paired *t* test, *p* < 0.001). At a steady-state holding potential of −20 mV, Na_V_1.7 channels activated for remarkably long durations producing a mean of 1735 ± 2495 ms (median, 897 ms, 93 individual activations from nine patches). At a steady-state holding potential of −110 mV, channel activity was briefer than at −20 mV, having a mean of 210 ± 270 ms (median, 98 ms, 336 individual activations from five patches, unpaired *t* test, *p* < 0.001). The intraactivation P_O_s were not different for channel activations between the two holding voltages. Channel activity recorded at −20 mV had an intraactivation P_O_ of 0.90 ± 0.09 (median, 0.92), and at −110 mV the P_O_ was 0.88 ± 0.12 (median, 0.91, *p* = 0.387) (Fig. 3*C*). No change to unitary current (∼1.5 pA at −20 mV) was observed during the Ta3a application period when the patches were continuously perfused with Ta3a-containing extracellular solution for up to 6 min.

These data suggest that Ta3a acts as a potent positive modulator of Na_V_1.7 channels, enhancing channel activity primarily by inducing long-lived active periods and diminishing channel inactivation, even at hyperpolarized voltages where channel activity would normally be absent.

### Ta3a binds more rapidly to activated Na_V_1.7 channels

Our experiments so far involved the continuous application of Ta3a while periodically activating the channels to monitor the effects of the peptide. This experimental protocol also rendered the channels constitutively active, even without subsequent depolarizing voltage steps. However, we also wished to test the hypothesis that channel activation facilitated Ta3a binding compared to channels that had not been subjected to prior voltage steps. To achieve this, currents were recorded from excised patches held at steady-state voltages of either −20 mV or −110 mV without applying any voltage steps to the patches. Prior to the application of Ta3a (and any applied voltage steps), the patches were silent at −20 mV and −110 mV. However, single channel activations became evident after prolonged exposure to 30 nM Ta3a at both holding voltages (Fig. 3, *D* and *E*). The mean peptide exposure time before channel activity appeared in the recordings was 20.0 ± 5.2 min, n = 5 patches.

The observation that Ta3a enhanced single channel active periods within 3 to 6 min when accompanied by activating voltage steps but required 20 min in the absence of activating voltage steps implies that Ta3a binds more readily to the active state of channels.

### Ta3a reduces single channel conductance after prolonged exposure

A diminished inward driving force for Na^+^, as inferred for the whole-cell I-V experiment should also manifest on a single-channel level. However, our initial single-channel measurements showed an unaltered single-channel amplitude upon continuous, direct perfusion with Ta3a-containing solution (Fig. 3, *A* and *B*). To investigate this inconsistency, we recorded single-channel activity using an alternative recording protocol. We exposed patches to Ta3a by direct perfusion and stepped the transmembrane voltage using the standard −110 mV to −20 mV steps to recapitulate our initial experiments, recorded single-channel currents at −20 mV, then turned off the perfusion. After a relatively prolonged period (∼20 min) in the absence of direct perfusion of the recorded patch, we re-recorded single-channel currents. From three patches that remained stable over the course of the experiment we measured the single channel amplitude in the control conditions to be −1.46 ± 0.05 pA and after ∼20 min to be −0.70 ± 0.17 pA (n = 3 patches, paired *t* test, *p* = 0.0018, Fig. 3, *F* and *G*). These data show that the reduced driving force is also observable on a single-channel level.

### Origin of changes to V_rev_ and V_1/2_ in Ta3a-exposed Na_V_1.7 channels

Reversal potentials can be affected by perturbations to the ion permeation pathway that alter the permeability properties of channels, such as pore-binding ligands (15, 17) or pore mutations (24, 25). Alternatively, changes in reversal potential can reflect changes in the ionic concentration gradients of permeant ions across the cell membrane (25). These two alternatives are manifest in the ionic concentration (or activity) terms and the relative permeability coefficients in the GHK equation that models the key driving forces of selective ion permeation (*i.e.*, Equations 2 and 3) (25). We investigated both factors separately using whole-cell electrophysiology.

**Figure 4:**
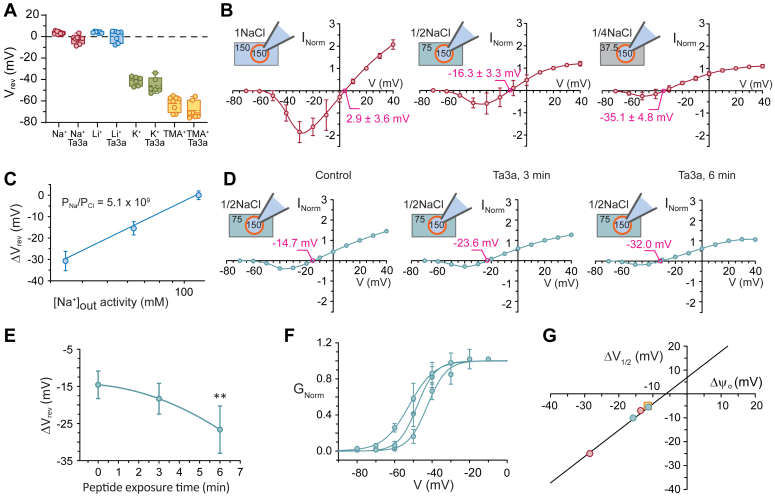
**Ta3a effects on ionic selectivity, reversal potential, and surface potential.***A*, bi-ionic reversal potential measurements in control (Na^+^) and test cations (Li^+^, K^+^, and TMA^+^) in the presence and absence of Ta3a. The Ta3a measurements were taken after 3 to 5 min of Ta3a perfusion (n = 6–11 cells). Cation pairs were compared using unpaired *t* tests. *B*, reference dilution potential measurements where the extracellular NaCl was diluted from a symmetrical 150 mM to a half dilution of 75 mM and a quarter dilution of 37.5 mM (n = 7 cells). *C*, group data of changes in reversal potential (ΔV_rev_) as a function of extracellular Na^+^ ion activity. The data were fit to the Goldman-Hodgkin-Katz (Equation 1) to obtain the relative Na^+^ to Cl^−^permeability ratio (P_Na_/P_Cl_). *D*, Ta3a measurements from an example cell with a constant extracellular solution of 75 mM NaCl in control and after 3 min and 6 min of perfusion of Ta3a (30 nM). Note the exposure time-dependent leftward shift in the I-V in Ta3a. *E*, group plot of the shift in V_rev_ as a function of continuous perfusion of Ta3a. ∗∗*p* < 0.005 (one-way ANOVA, n = 5 cells). *F*, conductance-voltage plots showing the exposure time-dependent leftward shift in V_1/2_ in Ta3a affected cells in extracellular solution of 75 mM NaCl. *G*, plot of the change in V_1/2_ as a function of a change in external surface potential, $\psi_{O}\text{.}$ The V_1/2_ values were determined from conductance-voltage plots of the reference dilution potential experiments shown in (*B*) (*red* data points), the Ta3a affected experiments in (*D* and *E*) (75 mM NaCl, *blue* data points) and from 150 mM NaCl (*yellow* data point, n = 3 cells). The data were fitted to Equation 5 and demonstrated that the changes in V_1/2_ correlate to changes in external surface potential. TMA, tetramethylammonium.

To determine if the observed changes to V_rev_ in the presence of Ta3a could be due to the peptide binding within, or near enough to the pore to alter ion selectivity, as has been reported for batrachotoxin (15, 17), we performed bi-ionic experiments to determine the lithium-to-sodium (P_Li_/P_Na_), potassium-to-sodium (P_K_/P_Na_) and tetramethyl ammonium-to-sodium (P_TMA_/P_Na_) permeability ratios in the absence and presence 30 nM Ta3a (Fig. 4*A*). In control experiments (no Ta3a), the mean reversal potentials for the I-Vs that were recorded in symmetrical NaCl was 3.3 ± 1.3 mV (n = 11 cells). Cells that were perfused with Ta3a reversed at −2.7 ± 3.6 mV (ΔV_rev_ = −6.0 mV). Perfusing cells with KCl produced a control V_rev_ of −41.5 ± 4.2 mV (n = 6 cells) and a V_rev_ in Ta3a of −45.8 ± 2.3 mV (ΔV_rev_ = −4.3 mV). Cells perfused with LiCl had a reversal potential of 4.0 ± 0.9 mV (n = 7 cells) in the absence of Ta3a and 0.3 ± 2.8 mV when perfused with Ta3a (ΔV_rev_ = −4.3 mV). Control TMACl produced a V_rev_ of −62.4 ± 7.0 mV (n = 7 cells) and in Ta3a the V_rev_ was −68.8 ± 8.0 mV (ΔV_rev_ = −6.4 mV, Fig. 4*A*). Using Equation 3, a P_Cl_/P_Na_ of zero (see below) and the activity of extracellular Na^+^ in the test solution (${a_{Na}}_{o}^{T}$) of zero, we obtained a control P_K_/P_Na_ of 0.17, a control P_Li_/P_Na_ of 0.98 and a control P_TMA_/P_Na_ of 0.07. Ta3a-induced leftward shifts in reversal potential (∼−4–7 mV) after 2 to 3 min of peptide exposure, but this occurred for the I-Vs in the Na^+^ control solutions and all test cations, and they were not statistically different (unpaired *t* test, all *p* > 0.05) from their respective Ta3a-free controls. The calculated permeability ratios relative to Na^+^ for K^+^, Li^+^ and tetramethylammonium (TMA^+^) were 0.19, 1.07, and 0.07, respectively in the presence of Ta3a. These data suggest that Ta3a made no appreciable change to ionic selectivity of the channels and is unlikely to interact with the selectivity filter of the pore.

To probe if ionic gradients might affect the observed V_rev_ values, we first carried out a set of reference experiments where we deliberately altered the transmembrane ion concentration gradient by successively decreasing the extracellular NaCl concentration. Changes in V_rev_ were monitored upon incremental dilutions of extracellular NaCl in the absence of Ta3a and here too, we used solutions that contained only Na^+^ and Cl^−^ as potential current carrying ions on both sides of the cell membrane (24, 25). In near symmetrical NaCl (∼150 mM) solutions the I-V reversed at a potential of +2.9 ± 3.6 mV (n = 7 cells, Fig. 4*B*). Progressive dilutions of the extracellular solution to 75 mM, and then to 37.5 mM produced corresponding leftward shifts to the reversal potential of −16.3 ± 3.3 mV and −35.1 ± 4.8 mV, respectively in accordance with the equilibrium potential for Na^+^ ions. After correcting for liquid junction potentials, the shifts in reversal potential (ΔV_rev_) (0 ± 3.6 mV, −15.5 ± 3.3 mV and −30.7 ± 4.8 mV) were plotted as a function of extracellular Na^+^ activity and fitted to the GHK equation for Na^+^ and Cl^−^ ions (23, 24, 25) (Equation 2). This analysis produced a relative permeability ratio for Na^+^ *versus* Cl^−^ (P_Na_/P_Cl_) of 5.1 × 10^9^, suggesting negligible Cl^−^ permeation through Na_V_s (Fig. 4*C*). These data demonstrate that with decreasing extracellular Na^+^ concentration, the reversal potential tracks the equilibrium potential for Na^+^ ions. We also noted a negative shift in the voltage threshold for channel activation with successive NaCl dilutions, suggesting that the V_1/2_ shifts in the same direction as the V_rev_. The change in V_1/2_ (ΔV_1/2_) for the 1NaCl-1/2NaCl and 1NaCl-1/4NaCl were −7 mV and −25 mV, respectively. In addition, we observed a decrease in inward current in these I-Vs, consistent with the decrease in driving force for inward Na^+^ ion flux (Fig. 4*B*).

### Ta3a-affected Na_V_ channels reduce the local extracellular concentration of permeant Na ^+^ ions

We chose the half dilution of 75 mM extracellular NaCl as the control condition to further explore the effects of Ta3a on reversal potential. Our control (no Ta3a) I-Vs reversed at the expected reversal potential for this concentration gradient. After exposing the recorded cell to 30 nM Ta3a for 3 min the reversal potential shifted leftward. At a Ta3a exposure time of 6 min the reversal potential shifted further to the left. The mean reversal potentials were −14.6 ± 3.7 mV (control, 75 mM NaCl, n = 5 cells), −18.3 ± 4.2 mV (3 min), and −26.6 ± 6.4 mV (6 min, one-way ANOVA, *p* < 0.005 Fig. 4, *D* and *E*). Changes in V_1/2_ corresponding to 3 min and 6 min exposure times of Ta3a were −5.5 mV and −10.1 mV, respectively (Fig. 4*F*). The inward current also diminished as a function of Ta3a exposure time, consistent with a reduction in inward driving force for Na^+^ ions (Fig. 4*D*). We performed a similar experiment with the 150 mM NaCl extracellular concentration in the presence of 30 nM Ta3a for 3 min. These experiments also produced a negative shift in V_rev_, yielding values of 5.0 ± 3.6 mV (control) and −6.3 ± 3.8 mV in the presence of Ta3a (ΔV_rev_ = −11.3 mV, n = 3 cells) and a hyperpolarizing shift in V_1/2_ of −5.0 mV.

These data suggest that the reversal potentials and V_1/2_ of Na_V_1.7 channels that are exposed to Ta3a progressively shift toward more negative values and mirror the reference experiments where extracellular NaCl was progressively diluted. The inferred dilution of extracellular Na^+^ in the presence of Ta3a is also consistent with the diminished inward currents and the leftward shifts in V_rev_ in the presence of other cations (Fig. 4*A*).

### Ta3a affects the extracellular surface potential to reduce the voltage threshold for Na_V_1.7 activation

Our data are consistent with Ta3a affecting the extracellular surface potential ($\psi_{O}$) of the cell membrane and reducing the voltage threshold for channel activation (23, 26, 27, 28). To test this inference, we first plotted the change in V_1/2_ against the change in $\psi_{O}$ that resulted from the extracellular NaCl dilutions in our reference experiments, using Equation 4 to calculate $\psi_{O}$ and conductance-voltage plots to determine V_1/2_. These data produced the predicted linear relationship between V_1/2_ and $\psi_{O}$ as modeled by Equation 5 (29). Changes in $\psi_{O}$ in the presence of Ta3a that were carried out in 75 mM extracellular NaCl were determined by estimating the Na ^+^ activity at the membrane surface by extrapolation from the ΔV_rev_ *versus* extracellular Na ^+^ activity plot (Fig. 4*C*) and changes in V_rev_ from the relevant I-Vs and conductance-voltage plots derived from them (Fig. 4*F*). The changes to V_1/2_ obtained from these plots as a function of the calculated change in $\psi_{O}$ are shown in Figure 4*G* at 3 min and 6 min exposure to 30 nM Ta3a along with data for 3 min exposure to Ta3a in symmetrical (150 mM) NaCl. As is clearly demonstrated by the linear fit to these data (r^2^ = 0.9976), changes to V_1/2_ in the presence of Ta3a are closely correlated with changes to $\psi_{O}$ with a fitted slope close to 1, as predicted by Equation 5.

This analysis clearly demonstrates that the leftward shifts to V_1/2_ in the presence of Ta3a are in response to induced changes to $\psi_{O}$ and strongly imply that Ta3a affects the local concentration of ions at the outer membrane surface.

### The Ta3a[E24A + K25A] analog affects V_1/2_ and V_rev_ at elevated concentrations

**Figure 5:**
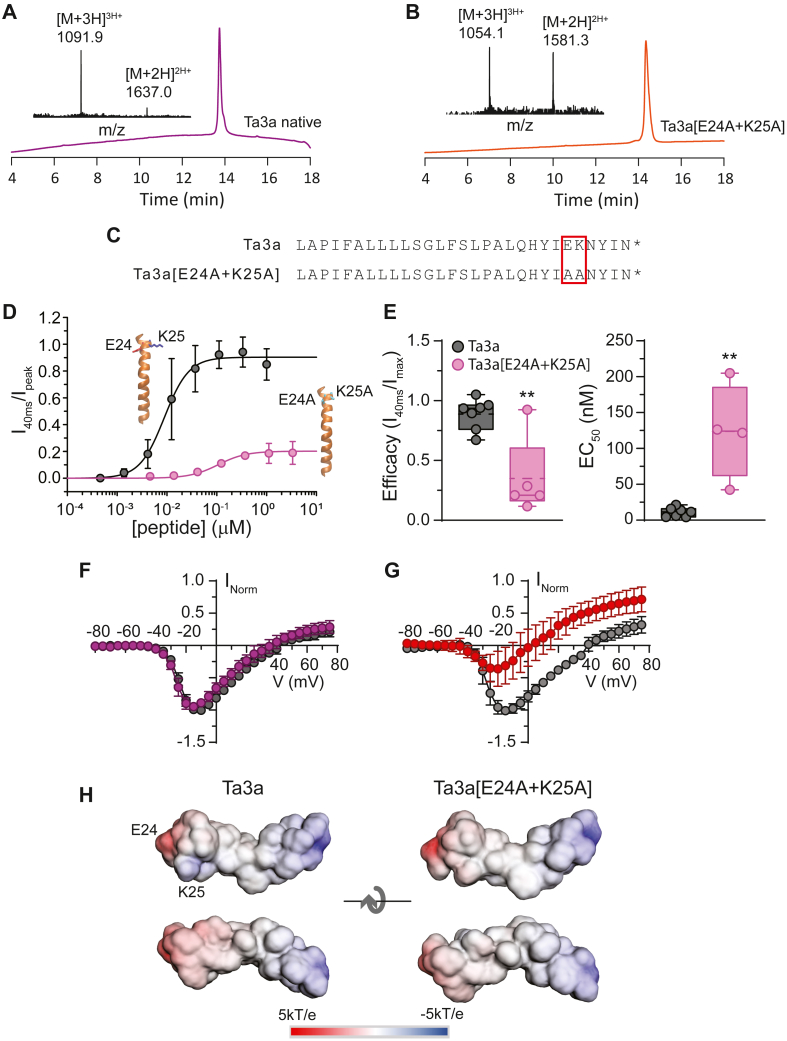
**Ta3a and Ta3a[E24A + K25A] analog.** High-resolution mass spectrum and HPLC trace of the native Ta3a (*A*) and Ta3a[E24A + K25A] analog (*B*). Flow rate (0.5 ml/min) with gradient 0 to 80% G in 16 min. Wavelength 214 nm. *C*, sequence of Ta3a and the Ta3a[E24A + K25A] analog showing the position of the two adjacent charges that were mutated to alanine in the analog. *D*, concentration-response plot of Ta3a (*black*) and the Ta3a[E24A + K25A] analog (*pink*) showing that the double mutation reduced the efficacy (Imax at saturation) and potency (EC_50_) at inducing non-inactivating current at Na_V_1.7 channels. *E*, Box and whisker plots of the efficacy of Ta3a and Ta3a[E24A + K24A] at inducing non-inactivating currents at Na_V_1.7 channels. *D*, Box and whisker plots of EC_50_ of Ta3a and Ta3a[E24A + K24A] at inducing non-inactivating currents at Na_V_1.7 channels. ∗∗*p* < 0.005 (unpaired *t* test), n = 4 to 7 cells. *F*, current-voltage plot of the Ta3a[E24A + K25A] analog in the presence (*dark pink*) and absence (*black*) of 30 nM Ta3a showing the loss of potency at leftward shifting V_1/2_, V_rev_ and reducing peak current (n = 4 cells). *G*, a similar experiment as in F but with 1 μM Ta3a[E24A + K25A] analog (*red*) showing that at a higher concentration the analog can reproduce some of the I-V alternations observed with Ta3a (n = 7 cells). *H*, surface potential estimates of Ta3a and the Ta3a[E24A + K25A] analog. Both peptides have a similar and weak helical dipole. The presence or absence of the two adjacent charges on Ta3a contributes little to the overall surface potential of the peptide.

Although Ta3a has a net neutral charge, it is conceivable that some of the charged residues might contribute to the apparent changes to $\psi_{O\;}$, or other effects on Na_V_1.7 function. To scrutinize if key charged residues of Ta3a contribute to the observed effects on Na_V_1.7 channels, we generated a double mutant analog of native Ta3a (Fig. 5, *A* and *B*) where the two adjacent charged residues near the C-terminal end of the peptide were substituted for alanine (E24A + K25A) (Fig. 5*C*). This was done to investigate if these charged residues affected the V_rev_, the V_1/2_ and the potency or efficacy with which the peptide induced persistent (non-inactivating) current. Cumulative whole-cell concentration-response experiments for native Ta3a and the double mutant analog demonstrated that potency and efficacy with which the analog induced persistent current was markedly reduced (Fig. 5*D*). The EC_50_ for native Ta3a was 11.1 ± 6.5 nM (n = 7 cells), whereas for the mutant analog the EC_50_ was 123.8 ± 6.6 nM (unpaired *t* test, *p* = 0.001, n = 4 cells, Fig. 5*E*). The efficacies between Ta3a and Ta3a[E24A + K25A] were 0.89 ± 0.13 and 0.35 ± 0.33 (unpaired *t* test, *p* = 0.003, Fig. 5*E*).

Nonetheless, despite the loss of potency, the double mutant peptide still affected changes to the V_rev_ and V_1/2_. Specifically, to account for the loss of potency of the analog we carried out I-V measurements at equipotent peptide concentrations (30 nM for native Ta3a, and 1 μM for Ta3a[E24A + K25A]. The I-V profile in the presence of the peptide analog at 30 nM was indistinguishable from control (Fig. 5*F*, n = 4 cells), in contrast to the effect of the WT peptide at 30 nM (Fig. 1*D*). However, at a concentration of 1 μM, the peptide analog produced a ∼2-fold reduction in peak current and leftward shifted the V_rev_ by −28 mV and the V_1/2_ by −4.5 mV (Fig. 5*G*, n = 7 cells).

These data suggest that E24 and K25 are important for interaction with the channel, but do not directly affect $\psi_{O}$. To further distinguish between these possibilities, we used the online Advanced Poisson-Boltzmann Solver (APBS-PDB2PQR; https://server.poissonboltzmann.org/) software suite (30) to evaluate the distribution of positive and negative surface potentials of the native and analog Ta3a peptides. The calculation produced the expected weak helix dipoles at the peptide termini, which is a feature of all helical peptides (31). Due to the proximity to each other and their opposite charge, the E24 and K25 residues made only minor contributions to the overall surface potential to Ta3a. A similar analysis of the potential of the double mutant analog corroborated this. Neutralizing the E24 and K25 left only the helix dipole, which produced a similar surface potential profile to the native Ta3a peptide. This suggests that the E24 and K25 residues likely shield each other and make negligible contributions to the overall charge of the peptide (Fig. 5H). This also suggests that the mutations would not affect interactions between the peptide analog and the cell membrane compared to native Ta3a. At elevated concentrations, (1 μM), the double mutant analog produced a clear leftward shift on V_rev_; however, the negligible differences in surface potential between the mutant analog and WT Ta3a, suggests that the charges are more likely important in peptide-channel interactions rather than peptide-ionic gradient effects, *per se*.

## Discussion

Our data are consistent with Ta3a altering the extracellular membrane surface potential ($\psi_{O}$) to reduce the activation threshold of Na_V_ channels. Changes to $\psi_{O}\text{,}$ and the resulting shift in the activation threshold for Na_V_ channels have been observed in response to extracellularly applied Ca^2+^ and H^+^ ions (23, 26). Natural adaptation in voltage-gated channel amino acid sequences have provided additional evidence that charges at or near the extracellular and intracellular surfaces of the channels affect surface charge and the activation properties of the channels (27, 28, 32). The effects we observe with Ta3a at Na_V_ channels are predicted by the Gouy-Chapman-Stern charge screening model, which consists of a bilayer of fixed charges embedded in the outer membrane surface and mobile counterions, which give rise to a negative change in $\psi_{O}$ (33, 34). A negative change in $\psi_{O}$ would affect the electric field within the cell membrane, which would be sensed by intramembrane Na_V_ channel voltage sensors (S4 domains) as equivalent to an externally applied transmembrane depolarization. Our analysis of the surface charge of Ta3a suggests that the peptide is overall neutral and not likely the source of the fixed positive charge that would electrostatically attract the mobile negative charges to the membrane surface. We propose that Ta3a binds to Na_V_ channels and stabilizes the S4 domains in the activated (up) configuration as has been proposed for β-scorpion toxins (19). As each of the S4 gating domains carries at least four fixed positive charges, and Na_V_ channel activation requires the upward translocation of an estimated 12 to 16 gating charges per channel (10, 11, 12), we propose that the S4 domains are the source of the fixed outer layer of positive charge. The positively charged S4 domains would electrostatically repel cations such as Na^+^ to produce a local Na^+^ dilution effect at the membrane surface (leftward shift in V_rev_) and attract negative counterions (Cl^−^) that generate the negative surface potential (leftward shift in V_1/2_). Cells with a greater number of channels (greater charge density) would produce a more pronounced dilution of mobile cations and concentration of mobile anions at the membrane surface (34). Plotting changes in V_1/2_ as a function of changes in $\psi_{O}$ in the presence of Ta3a produced a linear correlation between these two parameters, illustrating that the activation threshold of the channels is determined by $\psi_{O}$, which strongly suggests a concentration gradient between the bulk solution and the membrane surface in the presence of Ta3a.

We noted small (∼−4–7 mV) but consistent negative shifts in the I-Vs of other cations, consistent with the repulsion of local mobile positive charges at the membrane surface. The process occurs more readily when the membrane is depolarized by voltage steps in the presence of Ta3a because this promotes Ta3a binding to channels, likely by making the binding site of Ta3a more accessible. We infer that Ta3a-affected channels provide sufficient changes to $\psi_{O}$ to reduce the activation threshold of neighboring Na_V_ channels that are not yet bound to Ta3a. We have demonstrated that the pool of channels that are yet to be affected by Ta3a retain normal inactivation kinetics (22). This produces the Ta3a exposure-time dependent waveform in our I-Vs consisting of a decreasing pool of Na_V_ channels that activate in response to applied voltage steps and a proportional increase in the noninactivating pool. The observed decrease in whole-cell peak current in our I-Vs is a product of an increase in noninactivating, Ta3a-bound channels that can no longer be activated by voltage steps. Moreover, the decrease in Na^+^ (or other permeant cations) at the outer membrane surface would further reduce inward current, producing an overall decrease in peak inward currents observed in our I-Vs and single channel recordings. Ta3a clearly differs both structurally and in its fundamental mechanism of action at Na_V_ channels compared to disulfide-rich peptides derived from arachnid venoms. Our data also demonstrate that Ta3a has similar effects at other Na_V_ channel subtypes and is likely to operate *via* similar molecular and cellular mechanisms as those proposed for the Na_V_1.7 channel. Indeed, other Ta3a-related peptides from other ant species that we have identified show comparable effects as those reported here (22) and may represent a group of peptides with a unique set of mechanistic actions at Na_V_ channels.

Our study has investigated the action of the ant venom peptide Ta3a at Na_V_ channels and discovered intriguing cellular and molecular mechanisms that are consistent with increased neuronal excitability and the intense and long-lasting pain induced by *T. africanum* stings (22). We show that Ta3a can recruit the intrinsic activation machinery of vertebrate Na_V_ channels to modulate them both directly and indirectly by affecting their ionic microenvironment.

## Experimental procedures

### Peptide synthesis

A Liberty Prime (CEM) automated microwave synthesizer was used to assemble Ta3a and analog at 0.1 mmol scale on Fmoc-Rink amide resin (RAPP Polymer, 0.72 mmol/g). Peptide sequence assembly was performed in dimethylformamide using 5 eq of Fmoc protected amino acid/5eq Oxyma Pure/10 eq N,N′-diisopropylcarbodiimide for 1 min at 105 °C. Fmoc protecting group removal was achieved in 25% pyrrolidine/dimethylformamide (40 s at 100 °C). Cleavage and simultaneous removal of sidechain protecting groups was achieved by treatment with 95% trifluoracetic acid (TFA)/2.5% triisopropylsilane/2.5% H_2_O for 2 h at room temperature. Following filtration of cleavage solution, ice-cold diethyl ether was added to precipitate the crude peptides. The crude peptides were centrifuged (5000 rpm for 5 min × 3) then lyophilized in 0.05% TFA/45% acetonitrile/H_2_O.

### Reversed-phase high-performance liquid chromatography (RP-HPLC)

Preparative and analytical HPLC were carried out on a Shimadzu LC-20AT system equipped with SPD-20A Prominence UV/VIS detector, and SIL-20AHT autoinjector. An Eclipse XDB–C18 column (Agilent Technologies; 10 μm, 21.2 cm × 250 mm, 300 Å, flow rate 20 ml/min) with gradient from 40% to 80% solvent B in 40 min was used to purify the peptides. A Hypersil GOLD–C18 column (Thermo Fisher Scientific; 3 μm, 2.1 × 100 mm, 175 Å, flow rate 0.5 ml/min) with gradient 0 to 80% B in 16 min was used to analyze the purity of the peptides. Absorbance was monitored at 214 nm and 280 nm. Solvent A: 0.05% TFA in H_2_O; solvent B: 90% acetonitrile/0.05% TFA in H_2_O.

Stock solutions of Ta3a were prepared by dissolving lyophilized peptide in 100% dimethyl sulfoxide and diluted to the desired concentration in extracellular solution with 0.005% (conventional patch clamp) or 0.1% (automated patch clamp) bovine serum albumin.

### Conventional patch clamp electrophysiology

All experiments were done at room temperature. HEK293 AD cells stably expressing human Na_V_1.7 channels along with the β1 and β2 accessory subunits (SB Drug Discovery) were seeded onto Poly-D-lysine coated, 12 mm coverslips two days prior to experiments. Patch electrodes were made from borosilicate glass capillaries (G150F-3; Warner Instruments) and heat-polished to a final resistance of 5 to 12 MΩ (single channel experiments) or 2 to 4 M Ω (whole-cell experiments) when filled with intracellular solution. Cells were mycoplasma-free for all experiments.

Single-channel recordings were made in the excised outside-out patch configuration. Currents were recorded with an EPC 10 USB Heka patch clamp amplifier (HEKA, Elektronik), filtered (−3 dB, four-pole Bessel) at 5 kHz, and sampled at 50 kHz using PatchMaster software (https://heka.com/). Currents were elicited by applying 20 to 50 1 s voltage steps to −20 mV from an initial holding voltage of −110 mV at a step frequency was 0.5 Hz. Alternatively, recordings were done at a continuous clamped potential of either −20 mV or −110 mV. The extracellular solution contained (in mM): 140 NaCl, 5  KCl, 2  CaCl_2_, 1  MgCl_2_, 10  Hepes, and 10  D-glucose, adjusted to pH 7.4 with NaOH. The intracellular solution consisted of (in mM): 145 CsCl, 2 CaCl_2_, 2 MgCl_2_, 10 Hepes, and 5 EGTA adjusted to pH 7.4 with CsOH.

Whole-cell current-voltage (I-V) recordings were recorded with an EPC 10 USB Heka patch clamp amplifier or an Axon 700B amplifier (Axon Instruments). Currents were filtered (−3 dB, 4-pole Bessel) at 2 kHz and sampled at 50 kHz. Currents were elicited by a series of 10 ms voltage steps from a holding voltage of −110 mV to −70 mV and then at 10 mV (or 5 mV) increments to a final level of +100 mV. Leak subtraction was done using a p/4 protocol (4 pulses at a delay of 100 μs). Cell responses were accepted for analysis if the series resistance was ≤ 12 MΩ and was compensated for by ≥ 60%. In some figures the I-Vs are shown over a more restricted range of −70 mV to +40 mV for clarity. The extracellular solution contained (in mM): 140 NaCl, 5  KCl, 1  MgCl_2_, 10  Hepes, and 10  D-glucose, adjusted to pH 7.4 with NaOH, whereas the intracellular solution contained (in mM) the following: 140 CsCl, 10 NaCl, 2 10 Hepes, and 5 EGTA adjusted to pH 7.4 with CsOH.

Selected I-Vs including dilution potential and bi-ionic experiments were done using the voltage step protocol described above in simplified solutions (24). The extracellular 1NaCl solution comprised (in mM) the following: 145 NaCl, 10 Hepes, and 10 D-glucose. The half-diluted (1/2NaCl) extracellular solution contained (in mM) the following: 75 NaCl, 10 Hepes, 10 D-glucose, and 136 sucrose whereas the quarter-diluted extracellular solution contained (in mM) the following: 37.5 NaCl, 10 Hepes, 10 D-glucose, and 189 sucrose. For the bi-ionic experiments, the 145 mM NaCl in the control extracellular solution (above) was replaced by one containing 145 LiCl or KCl or TMACl, which was pH adjusted to 7.4 with 1 M LiOH, KOH, or TMAOH, respectively. The additional cation from the hydroxide solution was included in the final concentration of the cation for calculations of permeability ratios. The intracellular solution for all dilution and bi-ionic experiments consisted of (in mM): 145 NaCl, 5 EGTA, 10 Hepes, and pH adjusted to 7.4 with NaOH. All reversal potential values were corrected for liquid junction potentials, and ionic activities were used in the calculations instead of concentrations, which were determined by plotting tabulated activity coefficients against the concentration of each ion (24).

### Conductance-voltage analysis

Whole-cell conductance (G) was calculated using the following relation.

(1) $$G=\frac{I}{\left(Vm-Vrev\right)}$$

Where, I is the peak current, V_m_ is the corresponding step voltage and V_rev_ is the reversal potential (potential where current direction changes).

### Calculating permeability ratios relative to Na^+^ (P_X_/P_Na_) and external surface potential ($\psi_{O}$)

We used the GHK equation (Equation 1) to estimate the P_Cl_/P_Na_ ratio. A modified version of the GHK equation (Equation 2) was used to determine the permeability ratios of K^+^, Li^+^, and TMA^+^ as previously described (24).

(2) Vrev=(RTF)ln[(aNa)o+(PClPNa)(aCl)i(aNa)i+(PClPNa)(aCl)o]

(3) ΔVrev=(RTF)ln[aNaoT+(PLiPNa)(aLi)o+(PClPNa)(aCl)iaNaoC+(PClPNa)(aCl)i]

Where V_rev_ is the reversal potential. *a*_*ion*_ is the activity of the ion in the extracellular (subscript *o*) or intracellular (subscript *i*) solution. P_X_/P_Na_ is the permeability of ion X relative to that of Na^+^.

The extracellular surface potential ($\psi_{O}$) and its relation to ion concentration is (23, 35),

(4) $$C_{1}={C_{2}e}^{\left(\frac{-zF\psi_{O}}{RT}\right)}$$

The relationship between V_1/2_ and $\psi_{O}$ is (29),

(5) $$V_{\frac{1}{2}}=\psi_{O}-\left(\frac{\Delta G_{0}}{F}+\psi_{i}\right)$$

Where *C*_*1*_ is the new (diluted) solution and *C*_*2*_ is the original (bulk) solution. For experiments in Ta3a *C*_*1*_ was determined by extrapolation from our reference ionic dilution experiments, Z is the valence of Na^+^ and R, T and F and have their usual thermodynamic definitions.

### Automated electrophysiology

The same cells that were used for conventional patch clamp experiments were also used for automated electrophysiology, which were cultured in minimum essential medium containing fetal bovine serum (10% v/v), 2 mM L-glutamine, and selection antibiotics as recommended by the manufacturer at 37 °C with 5% CO_2_. Cells were passaged every 3 to 4 days upon reaching 60 to 70% confluency. The potency and efficacy of Ta3a and the Ta3a[E24A + K25A] analog and the corresponding I-V experiments were determined using a QPatch16 using single-hole plates (QPlate 16 with standard resistance of 2.0 ± 0.4 MΩ). Whole-cell currents were acquired at 25 kHz and filtered at 8 kHz. Series resistance ranged between 5 and 10 MΩ across recorded cells and was compensated at 70%. Concentration-response curves were obtained using a holding potential of −90 mV and 50 ms pulses to −20 mV applied at an interval of 20 s (0.05 Hz) for Na_V_1.4, Na_V_1.5, Na_V_1.6 and Na_V_1.7 and to +10 mV for Na_V_1.8. I-V curves were obtained by stepping the voltage from a holding potential of −90 mV followed by a series of 50 ms step pulses from −110 mV to +80 mV at 5 mV increments (repetition interval 6 s). The extracellular solution comprised (in mM) the following: 70 NaCl, 70 choline chloride, 4 KCl, 1 MgCl_2_, 2 CaCl_2_, 10 glucose, and 10 Hepes, adjusted to pH 7.4 with NaOH and osmolarity to 305 mOsm with sucrose. The intracellular solution contained (in mM) the following: 140 CsF, 5 CsOH, 1 EGTA, 10 NaCl, and 10 Hepes, adjusted to pH 7.4 with CsOH and osmolarity to 320 mOsm with sucrose.

### Data analysis software and statistics

Data were plotted and analyzed using GraphPad Prism 10 (https://www.graphpad.com/features) or SigmaPlot 15 (https://grafiti.com/sigmaplot-detail/). Single-channel currents were analyzed using QuB software (https://qub.mandelics.com/), using a temporal resolution of 75 μs. Discrete single-channel active periods were defined by a nonconducting duration of 100 ms.

Statistical analysis and plotting were performed using SigmaPlot 15 or GraphPad Prism 10 software. All data are presented as mean ± SD, with n values representing the number of cells or patches. Data sets comprise ≥ 3 independent experimental measurements and were first tested for normal distribution prior to using paired or unpaired, two-tailed *t* tests as appropriate to determine statistical significance. Data that were not normally distributed were subjected to nonparametric, Wilcoxon (paired) or Mann-Whitney (unpaired) tests. In all experimental analyses, *p* < 0.05 was taken as the significance threshold.

## Data availability

The data supporting the findings of this study are contained within the manuscript. Data sets are available from the corresponding author upon reasonable request.

## Conflict of interest

The authors declare that they have no conflicts of interest with the contents of this article.
